# Quercetin enhances the probiotic and functional characteristics of indigenous *Lactiplantibacillus plantarum* SG1 isolated from raw cow milk

**DOI:** 10.1039/d6ra06744a

**Published:** 2026-09-08

**Authors:** Sumeyye Akbulut

**Affiliations:** a Department of Molecular Biology and Genetics, Faculty of Science, Ataturk University 25240 Erzurum Turkey sumeyya.arslan@atauni.edu.tr

## Abstract

The growing demand for functional foods has intensified interest in identifying indigenous probiotic strains with superior physiological properties and exploring natural compounds capable of enhancing their functionality. In the present study, the effects of quercetin on the probiotic-associated and functional characteristics of an indigenous *Lactiplantibacillus plantarum* SG1 isolated from raw cow milk were comprehensively investigated. The isolate was identified by phenotypic characterization and 16S rRNA gene sequencing. Quercetin was applied at minimum inhibitory concentration (MIC) and sub-inhibitory concentrations (1/2 MIC and 1/4 MIC), and its influence on probiotic performance was evaluated through acid and bile salt tolerance, simulated gastrointestinal survival, cell surface hydrophobicity, auto-aggregation, co-aggregation, antimicrobial activity, antioxidant capacity, and safety-related characteristics. The SG1 strain exhibited intrinsic probiotic potential, demonstrating high tolerance to acidic and bile conditions together with favorable surface properties and antimicrobial activity. Exposure to sub-inhibitory concentrations of quercetin, particularly at 1/2 MIC, significantly improved bacterial survival under acidic conditions and simulated gastrointestinal transit (*p* < 0.05). Quercetin supplementation also enhanced cell surface hydrophobicity, aggregation capacity, and antagonistic activity against foodborne pathogens while increasing DPPH and ABTS radical scavenging activities. The isolate showed no hemolytic, DNase, or gelatinase activities and displayed an acceptable antibiotic susceptibility profile consistent with probiotic safety requirements. Collectively, these findings demonstrate that quercetin acts as a physiological modulator capable of improving multiple probiotic-associated characteristics of *Lactiplantibacillus plantarum* SG1 without compromising bacterial viability. The synergistic interaction between quercetin and this indigenous strain highlights its potential application in the development of next-generation functional dairy products and probiotic formulations.

## Abstract 2

Sub-inhibitory quercetin, particularly at 1/2 MIC, enhanced gastrointestinal survival, surface properties, antimicrobial activity, and antioxidant capacity of indigenous *L. plantarum* SG1.

## Graphical abstract

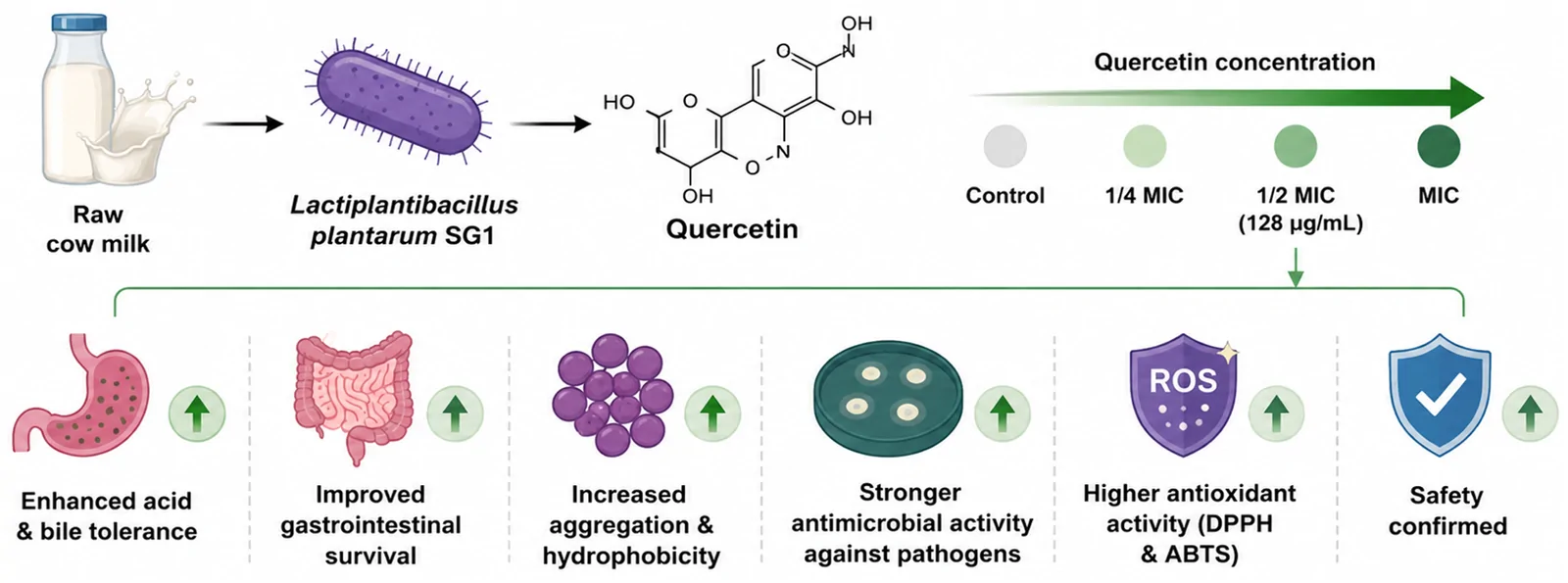

## 1. Introduction

Consumer interest in foods that provide health benefits beyond basic nutrition has accelerated the development of functional foods enriched with biologically active ingredients.^1,2^ Among these, probiotic microorganisms have received considerable scientific and industrial attention because of their ability to beneficially influence the intestinal microbiota and contribute to host health.^3,4^ According to the International Scientific Association for Probiotics and Prebiotics (ISAPP), probiotics are defined as live microorganisms that confer a health benefit on the host when administered in adequate amounts.^5^ Their beneficial effects extend beyond gastrointestinal colonization and include maintenance of intestinal barrier integrity, competitive exclusion of pathogens, modulation of immune responses, production of antimicrobial metabolites, and regulation of oxidative stress.^3,6^ Nevertheless, these beneficial activities are highly strain-dependent, emphasizing the importance of identifying and characterizing novel indigenous probiotic strains with superior functional properties.^7,8^

Among lactic acid bacteria, *Lactiplantibacillus plantarum* has emerged as one of the most versatile probiotic species because of its remarkable ecological adaptability and metabolic diversity.^9–11^ This species naturally occurs in a broad range of environments, including fermented foods, plant materials, and dairy products, enabling individual strains to develop unique physiological characteristics through adaptation to different ecological niches.^9,12^ Such adaptability contributes to improved tolerance against environmental stresses, including acidic conditions, bile salts, osmotic stress, and oxidative damage, all of which are essential attributes for successful gastrointestinal survival and probiotic functionality.^11,13^ Furthermore, numerous *L. plantarum* strains have demonstrated antimicrobial activity against foodborne pathogens, antioxidant capacity, cholesterol assimilation, and immunomodulatory properties, making them attractive candidates for functional food applications.^9–11^ However, because probiotic performance varies considerably at the strain level, each newly isolated strain requires comprehensive evaluation before industrial application.^7,14^ Raw cow milk represents an important natural reservoir of genetically diverse lactic acid bacteria.^8,12^ Unlike industrial starter cultures, indigenous microorganisms isolated from raw milk have adapted to complex microbial ecosystems and fluctuating environmental conditions, potentially conferring enhanced technological robustness and probiotic performance.^14,15^ Consequently, the isolation and characterization of novel *L. plantarum* strains from raw milk may provide valuable candidates for the development of innovative probiotic cultures with improved functional characteristics.^7,8^ Exposure to quercetin has been associated with improved bacterial tolerance to environmental stress, enhanced cell surface characteristics, increased antioxidant activity, and modulation of antimicrobial metabolite production in selected lactic acid bacteria.^16,17^ Nevertheless, these responses appear to be highly strain-specific, and limited information is available regarding the interaction between quercetin and newly isolated indigenous *L. plantarum* strains.^10,18^ Despite the increasing number of studies investigating probiotic–polyphenol interactions, comprehensive evaluations integrating gastrointestinal tolerance, cell surface characteristics, antimicrobial capacity, antioxidant activity, and safety assessment within a single indigenous strain remain scarce.^7,18^ Furthermore, most previous investigations have focused on commercial probiotic cultures rather than naturally occurring isolates obtained from raw dairy sources.^8,12,14^ Consequently, the extent to which quercetin can improve the probiotic performance of indigenous *Lactiplantibacillus plantarum* strains isolated from raw cow milk remains largely unexplored.^16,18^ Therefore, the present study aimed to comprehensively evaluate the influence of quercetin on the probiotic-associated and functional characteristics of an indigenous *Lactiplantibacillus plantarum* SG1 isolated from raw cow milk. The effects of quercetin were investigated using minimum inhibitory and sub-inhibitory concentrations by assessing acid and bile tolerance, simulated gastrointestinal survival, cell surface hydrophobicity, aggregation properties, antimicrobial activity, antioxidant capacity, and safety-related characteristics.^7,13,14^ It was hypothesized that quercetin would act as a physiological modulator capable of enhancing the probiotic performance of *L. plantarum* SG1, thereby supporting its potential application in next-generation functional dairy products and probiotic formulations.^9,16,18^

## 2. Materials and methods

### 2.1. Chemicals and reagents

All analytical-grade chemicals and microbiological media used throughout this study were purchased from commercial suppliers and prepared according to the manufacturers' instructions. Quercetin (≥95% purity), 2,2-diphenyl-1-picrylhydrazyl (DPPH), 2,2′-azinobis-(3-ethylbenzothiazoline-6-sulfonic acid) (ABTS), pancreatin, pepsin, oxgall bile salts, Mueller–Hinton agar (MHA), de Man, Rogosa and Sharpe (MRS) broth and agar, phosphate-buffered saline (PBS), and all other analytical reagents were obtained from Sigma-Aldrich (St. Louis, MO, USA), Merck (Darmstadt, Germany), or Oxoid (Basingstoke, UK). Fresh culture media were sterilized by autoclaving at 121 °C for 15 min before use. Quercetin stock solutions were freshly prepared in dimethyl sulfoxide (DMSO) immediately before each experiment and subsequently diluted with sterile MRS broth to obtain the desired working concentrations. The final DMSO concentration never exceeded 1% (v/v). A DMSO-only vehicle control matched to the solvent concentration of the quercetin-treated groups was not included in the original experimental design. Therefore, a solvent contribution to the observed responses cannot be completely excluded and is acknowledged as a methodological limitation.

### 2.2. Collection of raw cow milk samples

A total of twelve raw cow milk samples were aseptically collected from small-scale dairy farms located in Erzurum city center and the surrounding rural districts and villages of Erzurum Province, Türkiye. Sample collection was carried out during the same production season to minimize seasonal variations in the indigenous microbiota. Approximately 250 mL of raw milk was collected from each farm into sterile polypropylene containers immediately after milking. Samples were transported to the Food Microbiology Laboratory under refrigerated conditions (4 ± 1 °C) in insulated containers and processed within 4 h of collection to preserve the viability and diversity of indigenous lactic acid bacteria.^19^ The cow breed was not recorded at the time of sampling; therefore, breed-specific information cannot be reported retrospectively.

### 2.3. Isolation of lactic acid bacteria

Raw milk samples were homogenized by gentle inversion, and serial decimal dilutions (10^−1^ to 10^−7^) were prepared using sterile physiological saline (0.85% NaCl). Aliquots (100 µL) from appropriate dilutions were spread onto de Man, Rogosa and Sharpe (MRS) agar plates and incubated anaerobically at 37 °C for 48 h using AnaeroGen™ anaerobic atmosphere generation sachets (Oxoid, UK) in sealed anaerobic jars.

Following incubation, colonies displaying distinct morphological characteristics, including differences in size, color, margin, elevation, and surface appearance, were randomly selected and repeatedly streaked on fresh MRS agar until pure cultures were obtained. Purified isolates were maintained in MRS broth supplemented with 20% (v/v) glycerol at −80 °C for long-term preservation and routinely activated twice before experimental analyses.

### 2.4. Phenotypic characterization of lactic acid bacteria

Preliminary identification of presumptive lactic acid bacteria was performed based on colony morphology, Gram staining, catalase activity, cell morphology, motility, and spore formation according to the procedures described by Harrigan^20^ and Holzapfel and Wood.^21^ Only Gram-positive, catalase-negative, non-spore-forming, non-motile rod-shaped isolates were selected for further characterization.

Carbohydrate fermentation profiles were subsequently determined using the API 50 CHL identification system (bioMérieux, Marcy-l'Étoile, France) according to the manufacturer's recommendations. Fermentation patterns were interpreted using the APIweb™ software database to obtain preliminary species identification.

### 2.5. Molecular identification of *Lactiplantibacillus plantarum* SG1

The isolate demonstrating the most promising probiotic characteristics was designated SG1 and subjected to molecular identification by 16S rRNA gene sequencing. Genomic DNA was extracted from overnight cultures using a commercial bacterial genomic DNA purification kit according to the manufacturer's protocol. The nearly complete 16S rRNA gene was amplified using the universal bacterial primers *27F* (5′-AGAGTTTGATCMTGGCTCAG-3′) and *1492R* (5′-GGTTACCTTGTTACGACTT-3′). PCR amplification was performed in a thermal cycler under standard amplification conditions. Amplified PCR products were confirmed by electrophoresis on 1.5% agarose gels stained with ethidium bromide and visualized under UV illumination. Purified PCR products were commercially sequenced by Macrogen Inc. (Seoul, Republic of Korea). The resulting nucleotide sequences were assembled and compared with publicly available sequences deposited in the National Center for Biotechnology Information (NCBI) database using the Basic Local Alignment Search Tool (BLAST). Species identification was assigned according to the highest sequence similarity. The isolate was identified as *Lactiplantibacillus plantarum* and designated SG1 for all subsequent analyses.

### 2.6. Determination of the minimum inhibitory concentration (MIC) of quercetin

The minimum inhibitory concentration (MIC) of quercetin against *Lactiplantibacillus plantarum* SG1 was determined using the broth microdilution method according to the guidelines of Clinical and Laboratory Standards Institute^22^ and Wiegand *et al.*,^23^ with minor modifications adapted for lactic acid bacteria. Briefly, quercetin was dissolved in dimethyl sulfoxide (DMSO) to prepare a sterile stock solution and subsequently diluted with MRS broth to obtain a two-fold serial dilution ranging from 0.125 to 512 µg mL^−1^. An overnight culture of *L. plantarum* SG1 grown in MRS broth at 37 °C was adjusted to approximately 1 × 10^6^ CFU mL^−1^ using sterile saline. Aliquots of 100 µL of bacterial suspension and 100 µL of each quercetin dilution were dispensed into sterile 96-well microplates. Growth control wells containing bacterial suspension without quercetin and sterility control wells containing only culture medium were included in each assay. No DMSO-only vehicle control was included; consequently, solvent-specific effects were not independently quantified. Plates were incubated anaerobically at 37 °C for 24 h. Following incubation, bacterial growth was evaluated by measuring optical density at 600 nm using a microplate reader (BioTek Instruments, Winooski, VT, USA). The MIC was defined as the lowest quercetin concentration that completely inhibited visible bacterial growth compared with the untreated control. Based on the MIC value, 1/2 MIC and 1/4 MIC concentrations were selected for subsequent experiments to investigate the physiological effects of sub-inhibitory quercetin concentrations on probiotic characteristics.

### 2.7. Acid tolerance assay

Acid tolerance was evaluated to determine the ability of *Lactiplantibacillus plantarum* SG1 to survive under simulated gastric acidic conditions following exposure to quercetin. Overnight cultures were harvested by centrifugation (5000 × *g*, 10 min, 4 °C), washed twice with sterile phosphate-buffered saline (PBS), and resuspended in sterile MRS broth adjusted to pH 2.5 using 1 M hydrochloric acid. The bacterial suspensions were supplemented with quercetin at MIC, 1/2 MIC, or 1/4 MIC concentrations, whereas untreated cultures served as controls. Samples were incubated at 37 °C for 3 h under anaerobic conditions. Viable bacterial counts were determined immediately before and after acid exposure by serial dilution and spread plating on MRS agar. Plates were incubated anaerobically at 37 °C for 48 h, after which colonies were enumerated. Acid tolerance was expressed as the percentage survival relative to the initial bacterial population.

All experiments were conducted using three independent biological replicates, each analyzed in triplicate.

### 2.8. Bile salt tolerance

Bile salt tolerance was assessed to evaluate the ability of *L. plantarum* SG1 to survive under intestinal conditions. Fresh bacterial cultures were inoculated (1%, v/v) into sterile MRS broth supplemented with 0.3% (w/v) oxgall bile salts (Oxoid, UK), with or without quercetin at MIC, 1/2 MIC, and 1/4 MIC concentrations. Following anaerobic incubation at 37 °C for 4 h, viable cell counts were determined by serial dilution and plating on MRS agar. Survival percentages were calculated relative to bacterial counts obtained before bile exposure. The concentration of 0.3% bile salts was selected because it closely resembles the average physiological bile concentration encountered in the human small intestine and is widely accepted for evaluating probiotic tolerance.

### 2.9. Simulated gastrointestinal survival

The gastrointestinal survival of *L. plantarum* SG1 was evaluated using a sequential *in vitro* digestion model described by Charteris *et al.*^24^ simulating gastric and intestinal conditions. Initially, bacterial suspensions (approximately 10^8^ CFU mL^−1^) were transferred into simulated gastric fluid containing 3 g L^−1^ pepsin (Sigma-Aldrich) adjusted to pH 2.5 and incubated at 37 °C for 2 h under gentle agitation. Subsequently, cultures were centrifuged and resuspended in simulated intestinal fluid containing 1 g L^−1^ pancreatin and 0.3% oxgall bile salts, adjusted to pH 8.0, followed by incubation at 37 °C for an additional 3 h. Quercetin-treated groups received MIC, 1/2 MIC, or 1/4 MIC throughout the assay, while untreated cultures served as controls. Viable bacterial counts were determined after each digestion stage by serial dilution and plating on MRS agar. Overall gastrointestinal survival was expressed as the percentage of viable bacteria remaining after completion of the sequential digestion process.

### 2.10. Cell surface hydrophobicity

Cell surface hydrophobicity was determined using the microbial adhesion to hydrocarbons (MATH) method described by Rosenberg *et al.*,^25^ which estimates the affinity of bacterial cells for hydrophobic solvents and indirectly reflects their potential adhesion to intestinal epithelial surfaces. Overnight cultures were harvested by centrifugation, washed twice with PBS, and adjusted to an optical density of approximately 0.5 at 600 nm. Three milliliters of bacterial suspension were mixed with 1 mL of xylene by vigorous vortexing for 2 min and subsequently allowed to stand at room temperature for 30 min to facilitate phase separation. The aqueous phase was carefully collected, and absorbance was measured again at 600 nm. Cell surface hydrophobicity was calculated using the following equation:Hydrophobicity (%) = [1 − (*A*_1_/*A*_0_)] × 100where *A*_0_ represents the initial absorbance before xylene addition and *A*_1_ represents the absorbance of the aqueous phase after separation.

All measurements were performed in triplicate.

### 2.11. Auto-aggregation assay

The auto-aggregation capacity of *Lactiplantibacillus plantarum* SG1 was determined according to the method described by Del Re *et al.*,^26^ with slight modifications. Briefly, overnight cultures grown in MRS broth were harvested by centrifugation at 5000×*g* for 10 min at 4 °C and washed twice with sterile phosphate-buffered saline (PBS, pH 7.2). The bacterial pellets were subsequently resuspended in PBS to obtain an optical density of approximately 0.50 ± 0.02 at 600 nm (*A*_0_). The bacterial suspensions were vortexed for 10 s to ensure homogeneity and incubated at room temperature without agitation for 24 h. After incubation, 1 mL of the upper suspension was carefully removed without disturbing the sedimented cells, and the absorbance was measured again at 600 nm (*A*_*t*_).

Auto-aggregation percentages were calculated using the following equation:Auto-aggregation (%) = [1 − (*A*_*t*_/*A*_0_)] × 100where *A*_0_ represents the initial absorbance and *A*_*t*_ represents the absorbance after 24 h of incubation.

The assay was performed in triplicate for untreated cultures and cultures exposed to MIC, 1/2 MIC, and 1/4 MIC concentrations of quercetin.

### 2.12. Co-aggregation assay

The co-aggregation ability of *L. plantarum* SG1 with selected foodborne pathogenic bacteria was evaluated following the procedure described by Collado *et al.*,^27^ with minor modifications. Fresh cultures of *L. plantarum* SG1 and each indicator pathogen were harvested separately by centrifugation (5000 × *g*, 10 min), washed twice with PBS, and adjusted to an optical density of approximately 0.5 at 600 nm. Equal volumes (2 mL) of SG1 suspension and each pathogen suspension were mixed by gentle vortexing and incubated at room temperature for 4 h. Control tubes containing individual bacterial suspensions were prepared under identical conditions.

Following incubation, the absorbance of the mixed suspensions was measured at 600 nm. Co-aggregation percentages were calculated according to the following equation:Co-aggregation (%) = [(*A*_x_ + *A*_γ_ − 2*A*_mix_)/(*A*_x_ + *A*_γ_)] × 100where *A*_x_ and *A*_γ_ represent the absorbance values of the individual bacterial suspensions and *A*_mix_ represents the absorbance of the mixed bacterial suspension.

The assay was carried out against the pathogenic strains used throughout this study, and all experiments were performed in triplicate.

### 2.13. Antimicrobial activity

The antimicrobial activity of *Lactiplantibacillus plantarum*SG1 was determined using the agar well diffusion assay as previously described by Schillinger and Lücke,^28^ with slight modifications.

SG1 was cultivated in MRS broth at 37 °C for 24 h under anaerobic conditions. Cell-free culture supernatants (CFS) were obtained by centrifugation at 8000 × *g* for 10 min, followed by filtration through sterile 0.22 µm membrane filters to eliminate residual bacterial cells. Indicator pathogenic bacteria were grown overnight in appropriate culture media and adjusted to approximately 10^8^ CFU mL^−1^ (0.5 McFarland Standard). Sterile Mueller–Hinton agar plates were uniformly inoculated using sterile cotton swabs. Circular wells (6 mm diameter) were aseptically prepared in the agar, and 100 µL of sterile CFS obtained from untreated cultures and cultures exposed to MIC, 1/2 MIC, or 1/4 MIC concentrations of quercetin were individually dispensed into each well. Plates were maintained at 4 °C for 1 h to facilitate diffusion and subsequently incubated at 37 °C for 24 h. Antimicrobial activity was evaluated by measuring the diameter of the inhibition zones surrounding each well using a digital caliper. All measurements were recorded in millimeters (mm), excluding the well diameter. Each assay was performed in triplicate, and the results were expressed as the mean inhibition zone ± standard deviation.

### 2.14. Antioxidant activity

The antioxidant activity of *Lactiplantibacillus plantarum* SG1 was evaluated by determining the free radical scavenging capacity of cell-free culture supernatants (CFS) using the 2,2-diphenyl-1-picrylhydrazyl (DPPH) and 2,2′-azinobis-(3-ethylbenzothiazoline-6-sulfonic acid) (ABTS) radical scavenging assays. The antioxidant potential of untreated cultures and cultures exposed to sub-inhibitory concentrations of quercetin (MIC, 1/2 MIC, and 1/4 MIC) was comparatively assessed.

#### 2.14.1. DPPH radical scavenging assay

The DPPH radical scavenging activity of *L. plantarum* SG1 was determined according to the method described by Brand-Williams *et al.*,^29^ with slight modifications. Briefly, bacterial cultures grown under the respective experimental conditions were centrifuged at 8000 × *g* for 10 min at 4 °C, and the resulting supernatants were filtered through sterile 0.22 µm membrane filters to obtain cell-free culture supernatants (CFS). An aliquot of 1.0 mL of CFS was mixed with 1.0 mL of freshly prepared 0.2 mM DPPH solution in methanol. The reaction mixtures were vortexed thoroughly and incubated in the dark at room temperature for 30 min to allow complete radical reduction. After incubation, the absorbance was measured at 517 nm using a UV-Vis spectrophotometer (Shimadzu UV-1800, Kyoto, Japan). Methanol containing DPPH solution served as the control, while methanol without DPPH was used as the blank.

The DPPH radical scavenging activity was calculated according to the following equation:DPPH scavenging activity (%) = [(*A*_control_ − *A*_sample_)/*A*_control_] × 100where *A*_control_ is the absorbance of the control reaction and *A*_sample_ is the absorbance of the sample containing the bacterial supernatant.

All measurements were performed in triplicate using three independent biological replicates.

#### 2.14.2. ABTS radical scavenging assay

The ABTS radical scavenging activity was determined following the method of Re *et al.*,^30^ with minor modifications. The ABTS radical cation (ABTS˙^+^) was generated by reacting 7.0 mM ABTS solution with 2.45 mM potassium persulfate and allowing the mixture to stand in the dark at room temperature for 12–16 h before use. Prior to the assay, the ABTS˙^+^ solution was diluted with phosphate-buffered saline (PBS) to obtain an absorbance of 0.70 ± 0.02 at 734 nm. Subsequently, 200 µL of cell-free culture supernatant was mixed with 2.0 mL of diluted ABTS˙^+^ solution and incubated at room temperature for 6 min in the dark.

The decrease in absorbance was measured at 734 nm using a UV–Vis spectrophotometer. The radical scavenging activity was calculated using the following equation:ABTS scavenging activity (%) = [(*A*_control_ − *A*_sample_)/*A*_control_] × 100where *A*_control_ represents the absorbance of the ABTS radical solution without sample and *A*_sample_ represents the absorbance after reaction with the bacterial supernatant.

All experiments were conducted in triplicate, and antioxidant activity was expressed as the percentage inhibition of ABTS radicals.

### 2.15. Safety assessment of *Lactiplantibacillus plantarum* SG1

The safety characteristics of *Lactiplantibacillus plantarum* SG1 were evaluated by assessing hemolytic activity, DNase activity, gelatinase production, and antibiotic susceptibility. These analyses were performed to determine the suitability of the isolate for potential probiotic applications in accordance with internationally accepted safety recommendations for lactic acid bacteria proposed by FAO/WHO^31^ and EFSA.^32^

#### 2.15.1. Hemolytic activity

Hemolytic activity was evaluated using Columbia agar supplemented with 5% (v/v) defibrinated sheep blood. Fresh overnight cultures of *L. plantarum* SG1 were streaked onto blood agar plates and incubated anaerobically at 37 °C for 48 h. Following incubation, plates were visually examined for the presence of hemolytic zones surrounding bacterial colonies. Hemolytic reactions were classified as α-hemolysis (greenish discoloration), β-hemolysis (complete lysis with a transparent halo), or γ-hemolysis (absence of hemolysis). Only isolates exhibiting γ-hemolytic activity were considered suitable for subsequent probiotic evaluation. Positive and negative control strains were included to verify the accuracy of the assay.

#### 2.15.2. DNase activity

DNase production was investigated using commercially prepared DNase agar medium (Oxoid, UK). Activated cultures of *L. plantarum* SG1 were spot inoculated onto DNase agar plates and incubated anaerobically at 37 °C for 48 h. Following incubation, the plates were flooded with 1 N hydrochloric acid (HCl) to enhance visualization of DNA hydrolysis. The formation of clear halos surrounding bacterial colonies was interpreted as positive DNase activity, whereas the absence of a transparent zone indicated a negative reaction.

#### 2.15.3. Gelatinase activity

Gelatinase production was determined using nutrient gelatin medium according to the method described by Harrigan.^20^ Fresh cultures of *L. plantarum* SG1 were inoculated into sterile nutrient gelatin tubes and incubated at 37 °C for up to seven days. After incubation, the tubes were transferred to 4 °C for 30 min to evaluate gelatin liquefaction. Persistent liquefaction following refrigeration was interpreted as positive gelatinase activity, whereas complete solidification indicated the absence of gelatin hydrolysis.

#### 2.15.4. Antibiotic susceptibility

The antibiotic susceptibility profile of *L. plantarum* SG1 was determined using the Kirby–Bauer disk diffusion method following the recommendations of the Clinical and Laboratory Standards Institute.^22^ Briefly, overnight cultures were adjusted to approximately 0.5 McFarland turbidity standard and uniformly spread onto Mueller–Hinton agar plates supplemented with 5% defibrinated sheep blood to facilitate the growth of lactic acid bacteria. Commercial antibiotic discs (Oxoid, UK) were aseptically placed onto the agar surface using sterile forceps. Following anaerobic incubation at 37 °C for 24 h, inhibition zone diameters were measured using a digital caliper. The susceptibility profile of the isolate was interpreted according to CLSI recommendations and EFSA guidance for probiotic microorganisms. All assays were carried out in triplicate.

### 2.16. Statistical analysis

All experiments were conducted using three independent biological replicates, and each analysis was performed in triplicate. The results are presented as the mean ± standard deviation (SD). Statistical analyses were performed using IBM SPSS Statistics software (IBM Corp., Armonk, NY, USA). Differences among the control and quercetin-treated groups were evaluated using one-way analysis of variance (ANOVA). When significant differences were detected, Tukey's honestly significant difference (HSD) post hoc test was applied to compare the mean values among experimental groups. Prior to statistical analysis, data were examined for normal distribution and homogeneity of variance to verify compliance with the assumptions of ANOVA. Statistical significance was accepted at *p* < 0.05. All graphical representations were prepared using GraphPad Prism (GraphPad Software, San Diego, CA, USA).

## 3. Results

### 3.1. Isolation and identification of *Lactiplantibacillus plantarum* SG1 from raw cow milk

A total of twelve raw cow milk samples collected from traditional dairy farms located in Erzurum Province, Türkiye, were screened for the presence of indigenous lactic acid bacteria (LAB). Following cultivation on MRS agar under anaerobic conditions, several presumptive LAB colonies exhibiting distinct morphological characteristics were recovered. After repeated purification, representative isolates were subjected to comprehensive phenotypic, biochemical, and molecular characterization to identify a promising probiotic candidate. The selected isolate, designated SG1, exhibited typical characteristics of the genus *Lactiplantibacillus*, including Gram-positive, catalase-negative, non-spore-forming, non-motile, rod-shaped cells. Colonies grown on MRS agar were creamy-white, circular, convex, and smooth, consistent with the morphological characteristics commonly reported for *Lactiplantibacillus plantarum*. Preliminary biochemical identification using the API 50 CHL system demonstrated a carbohydrate fermentation profile characteristic of *L. plantarum*. Species identity was subsequently confirmed by sequencing of the nearly complete 16S rRNA gene using the universal primers 27F and 1492R. Sequence comparison against the NCBI database using the BLAST algorithm revealed a high sequence similarity with *Lactiplantibacillus plantarum*, confirming the phenotypic identification. The nucleotide sequence of isolate SG1 was deposited in the GenBank database under accession number PZ547809. Based on the combined phenotypic, biochemical, and molecular analyses, the isolate was conclusively identified as *Lactiplantibacillus plantarum* SG1. A comprehensive summary of the isolation procedure and identification results is presented in Table 1.

**Table 1:** Comprehensive characterization of the selected probiotic isolate (*Lactiplantibacillus plantarum* SG1) recovered from raw cow milk

Category	Parameter	Result
Isolation	Number of milk samples	12
	Sample source	Raw cow milk
	Geographical origin	Erzurum Province, Türkiye
	Selected isolate	SG1
Morphological characteristics	Colony morphology	Circular, creamy-white, convex, smooth
	Cell morphology	Rod-shaped
Physiological characteristics	Gram reaction	Positive
	Catalase activity	Negative
	Motility	Negative
	Spore formation	Negative
	Oxygen requirement	Facultatively anaerobic
Biochemical characterization	API system	API 50 CHL
	Species prediction	*Lactiplantibacillus plantarum*
Molecular characterization	Identification method	16S rRNA gene sequencing
	PCR primers	27F/1492R
	Amplicon size	∼1500 bp
	Sequencing provider	Macrogen Inc. (Seoul, Republic of Korea)
	Closest NCBI match	*Lactiplantibacillus plantarum*
	Sequence similarity	99.9–100%
	GenBank accession number	**PZ547809**
	Final identification	***Lactiplantibacillus plantarum* SG1**

### 3.2. Minimum inhibitory concentration of quercetin

The broth microdilution assay revealed that quercetin exerted a complete growth-inhibitory effect on *L. plantarum* SG1 at the highest tested concentration. The MIC value was determined as 256 µg mL^−1^. Based on this value, two sub-inhibitory concentrations 1/2 MIC (128 µg mL^−1^) and 1/4 MIC (64 µg mL^−1^) were selected for subsequent assays. Throughout all experiments, the final DMSO concentration never exceeded 1% (v/v). Because a matched DMSO-only vehicle control was not included, the independent contribution of the solvent cannot be excluded.

### 3.3. Acid tolerance

**Fig. 1:**
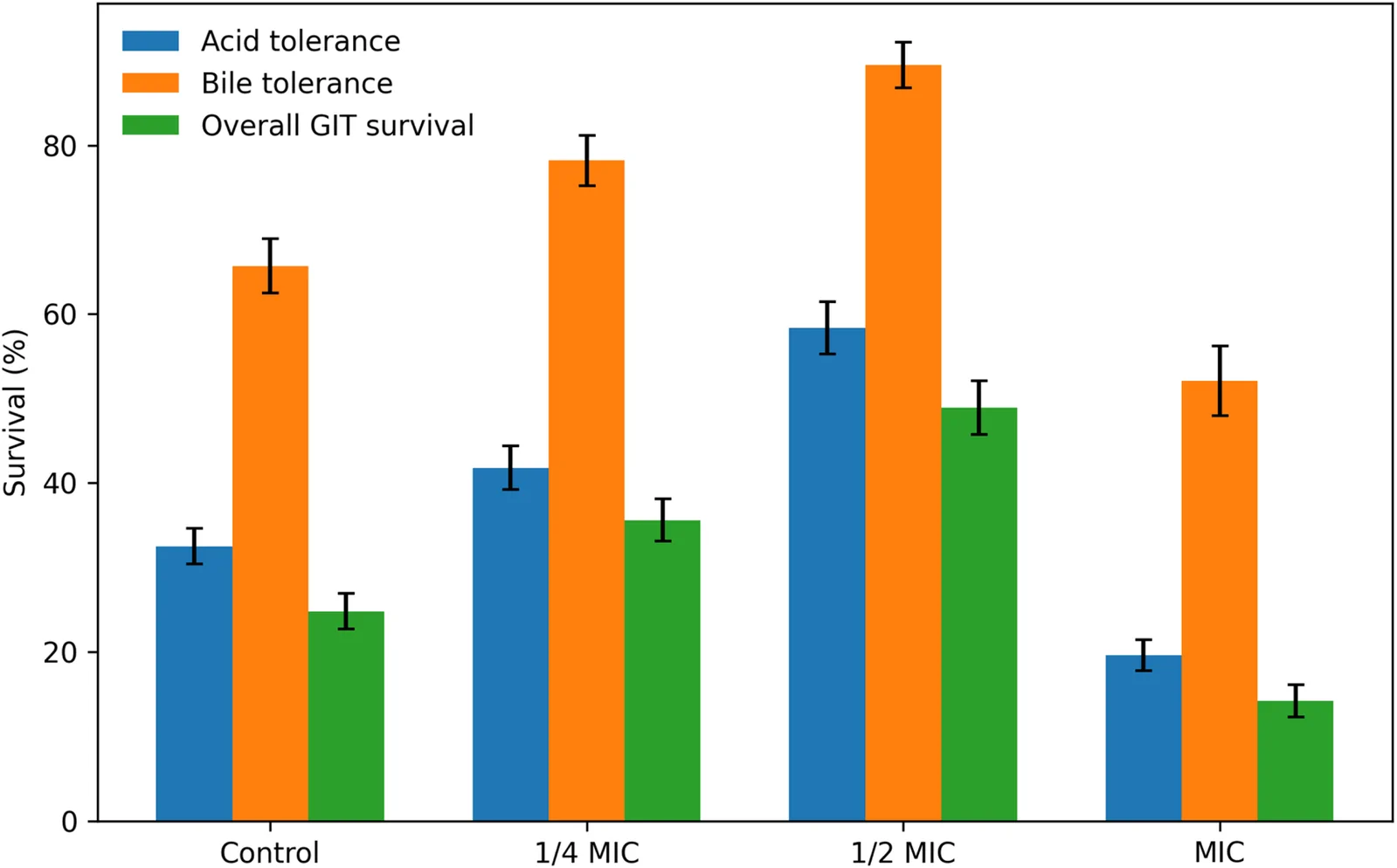
Effect of quercetin concentration on acid tolerance, bile salt tolerance, and overall simulated gastrointestinal survival of *L. plantarum* SG1. Bars represent mean ± SD.

The survival of *L. plantarum* SG1 under simulated gastric conditions (pH 2.5, 3 h, 37 °C) varied significantly among the experimental groups. As shown in Fig. 1 (numerical values are provided in Table S1 of the SI), untreated cells exhibited a survival rate of 32.5 ± 2.1%, whereas supplementation with quercetin at 1/4 MIC and 1/2 MIC significantly improved survival to 41.8 ± 2.6% and 58.4 ± 3.1%, respectively (*p* < 0.001). Exposure to MIC (256 µg mL^−1^), however, reduced survival to 19.6 ± 1.8%, confirming the bacteriostatic/bactericidal nature of quercetin at this concentration under acidic stress (Fig. 1). The present manuscript reports survival outcomes as percentages derived from viable plate counts. Replicate-level raw CFU mL^−1^ values were not retained in a form suitable for complete retrospective reporting; therefore, no raw CFU dataset has been reconstructed or inferred. This is acknowledged as a reporting limitation, and future studies should provide both absolute CFU mL^−1^ counts and percentage survival values.

### 3.4. Bile salt tolerance

The capacity of the strain to survive in the presence of 0.3% oxgall bile salts was also dose-dependently affected by quercetin (Fig. 1; Table S1, SI). The highest survival rate (89.5 ± 2.7%) was observed at 1/2 MIC, followed by 1/4 MIC (78.2 ± 3.0%), while the untreated control retained 65.7 ± 3.2% viability (Fig. 1). At MIC, survival dropped to 52.1 ± 4.1%, indicating that the inhibitory effect of quercetin on *de novo* membrane synthesis predominates in the absence of growth pressure.

### 3.5. Simulated gastrointestinal survival

Sequential exposure to simulated gastric and intestinal fluids produced a stepwise decline in viability, but sub-inhibitory quercetin markedly attenuated bacterial loss (Fig. 1; Table S1, SI). After the gastric phase, 1/2 MIC-supplemented cultures retained 62.3 ± 3.4% viability compared with 38.2 ± 2.9% for the untreated control (Fig. 1). After the intestinal phase, overall survival was highest in the 1/2 MIC group (48.9 ± 3.2%), which was approximately twice that of the control (24.8 ± 2.1%) (*p* < 0.001), confirming a cumulative beneficial effect.

### 3.6. Cell surface hydrophobicity

Cell surface hydrophobicity, assessed by the MATH assay using xylene, was substantially enhanced in the presence of sub-inhibitory quercetin. The highest hydrophobicity percentage (67.1 ± 2.8%) was obtained at 1/2 MIC, representing approximately 1.6-fold the value observed in the untreated control (42.3 ± 1.9%). The MIC treatment significantly decreased hydrophobicity to 31.5 ± 2.0% (*p* < 0.001) a finding consistent with reduced membrane integrity under bacteriostatic stress.

### 3.7. Auto-aggregation

Auto-aggregation percentage after 24 h of static incubation followed the same pattern. Sub-inhibitory quercetin (1/2 MIC) increased auto-aggregation to 51.2 ± 2.1%, *versus* 28.4 ± 1.5% in the untreated control (*p* < 0.001). A modest but significant increase was also detected at 1/4 MIC (38.6 ± 1.8%), while MIC suppressed this property to 18.7 ± 1.3%.

### 3.8. Co-aggregation with foodborne pathogens

**Fig. 2:**
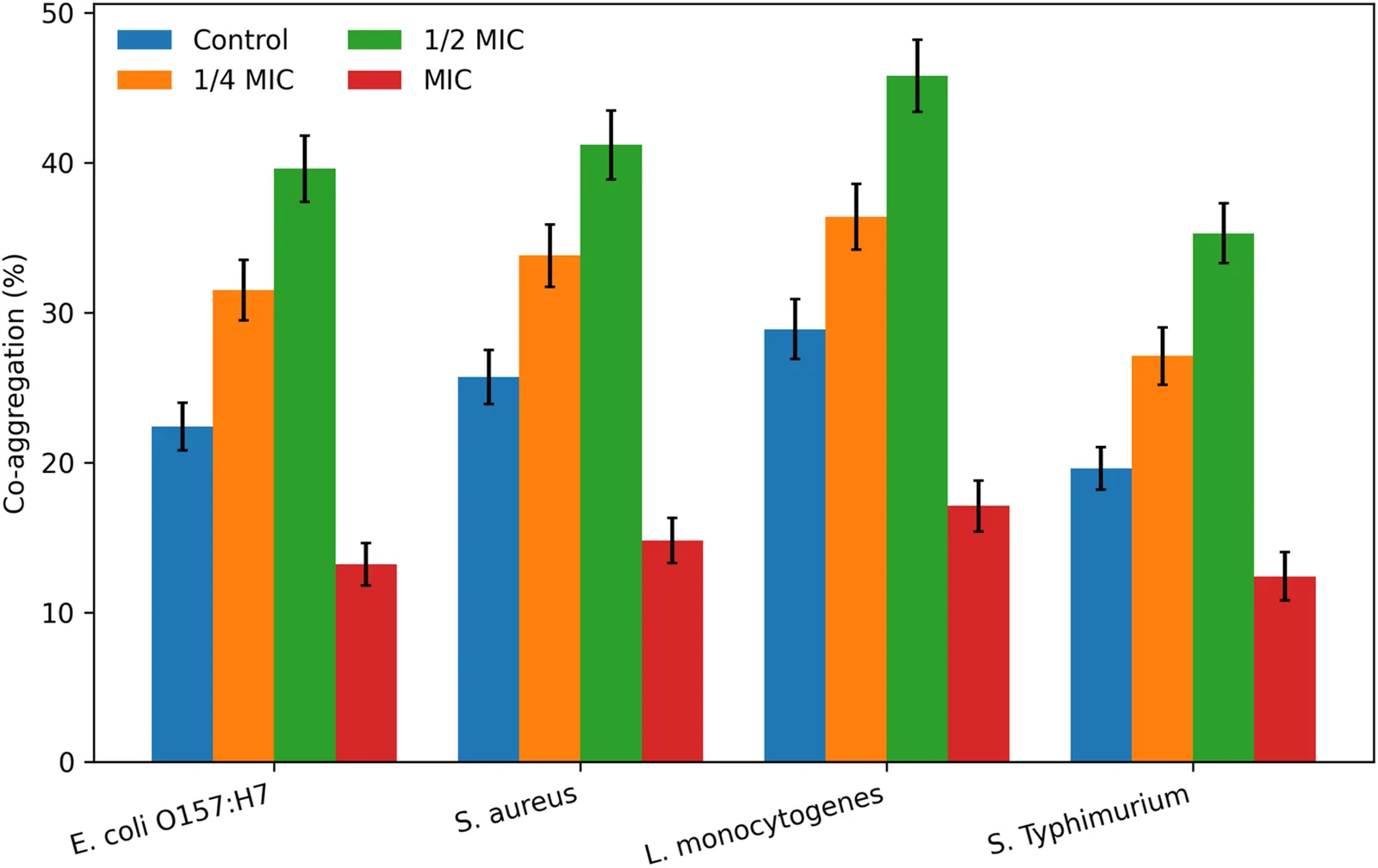
Effect of quercetin concentration on co-aggregation of *L. plantarum* SG1 with foodborne pathogens. Bars represent mean ± SD.

Co-aggregation of *L. plantarum* SG1 with four indicator pathogens varied according to both pathogen identity and quercetin dose (Fig. 2; Table S2, SI). The most pronounced co-aggregation was observed with *L. monocytogenes* at 1/2 MIC (45.8 ± 2.4%), whereas the lowest values were recorded for *Salmonella typhimurium* at MIC (12.4 ± 1.6%) (Fig. 2). One-way ANOVA confirmed a significant treatment effect for each pathogen pair (*p* < 0.001).

### 3.9. Antimicrobial activity

**Fig. 3:**
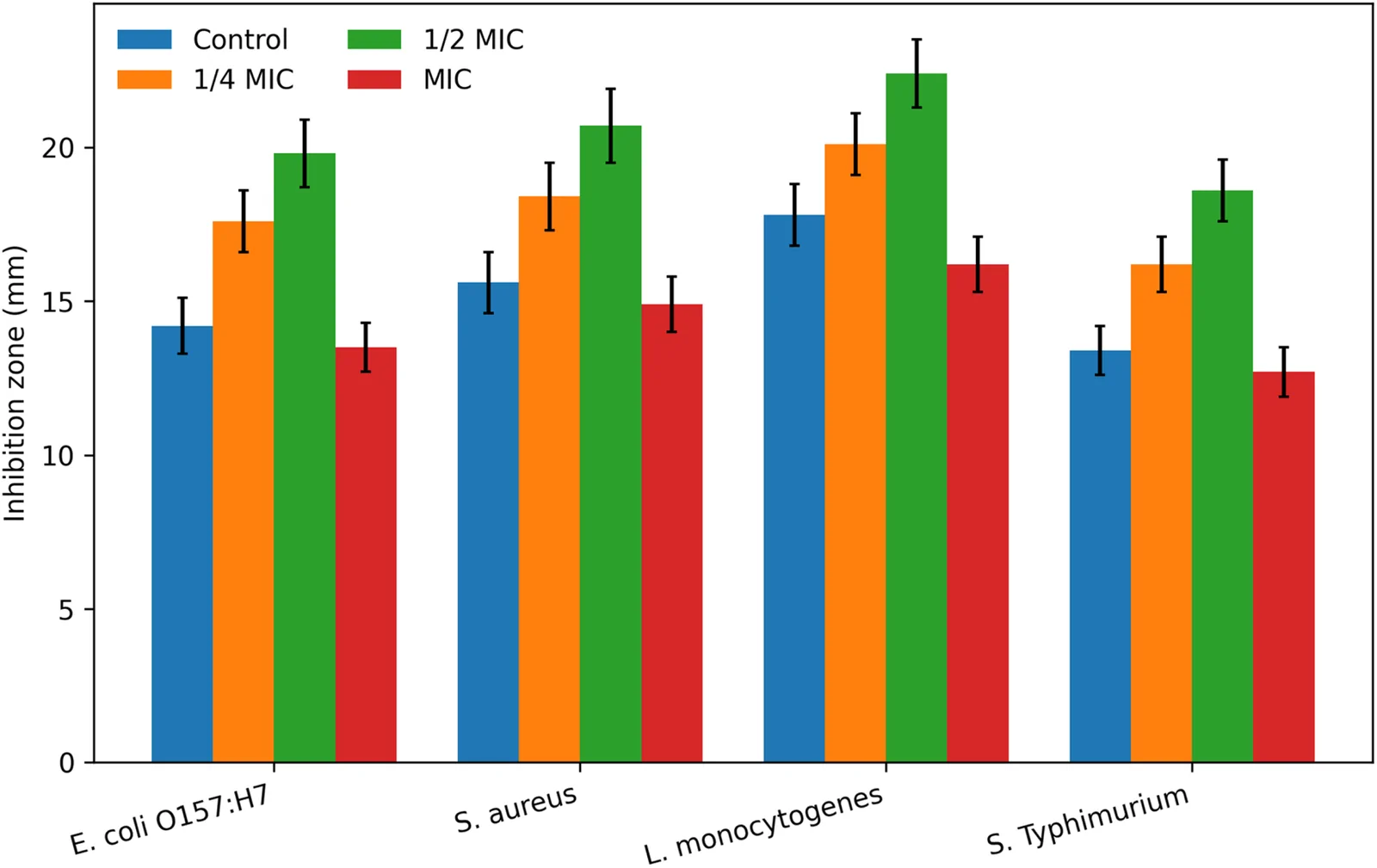
Antimicrobial activity of *L. plantarum* SG1 cell-free culture supernatants against foodborne pathogens after quercetin exposure. Bars represent mean inhibition zone ± SD.

Cell-free culture supernatants of *Lactiplantibacillus plantarum* SG1 inhibited all four pathogens tested, and inhibition zones were largest in the 1/2 MIC group (Fig. 3; Table S3, SI). The widest inhibition zone was observed against Listeria monocytogenes at 1/2 MIC (22.4 ± 1.1 mm) (Fig. 3). At MIC, antagonistic activity was comparable to the untreated control. Because the present study did not quantify individual antimicrobial metabolites or include a quercetin/DMSO-only diffusion control, the mechanism responsible for these differences cannot be assigned from the current data.

### 3.10. Antioxidant activity

Both DPPH and ABTS radical scavenging activities of the CFS were significantly enhanced by sub-inhibitory quercetin. DPPH inhibition increased from 38.7 ± 2.3% in the control to 52.4 ± 2.9% at 1/4 MIC and reached a maximum of 68.9 ± 3.4% at 1/2 MIC (*p* < 0.001). ABTS scavenging followed a parallel trend, with values rising from 42.1 ± 2.5% to 73.4 ± 3.6% at 1/2 MIC. Notably, at MIC, both activities declined to levels below the control, in line with reduced metabolic activity and metabolite secretion.

### 3.11. Safety assessment

**Fig. 4:**
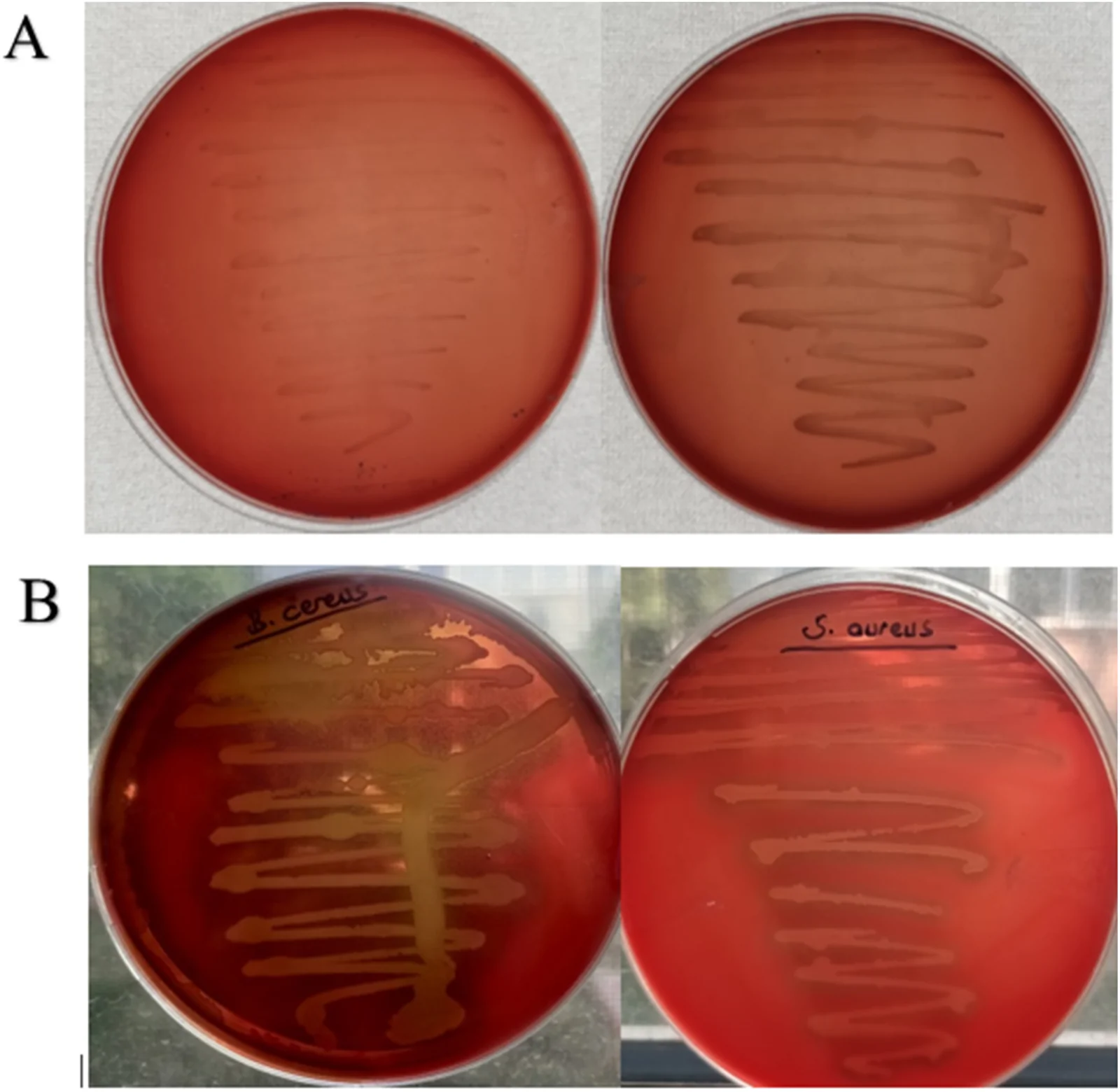
(A) γ-Hemolytic activity images of various isolates and (B) β-hemolytic activity images of *Bacillus cereus* and *Staphylococcus aureus* selected as positive control.

*L. plantarum* SG1 displayed a γ-hemolytic (non-hemolytic) phenotype on Columbia blood agar (Fig. 4), no DNase activity on DNase agar, and failed to liquefy nutrient gelatin after 7 days of incubation, indicating the absence of gelatinase production. The antibiotic susceptibility profile determined by the Kirby Bauer disk diffusion method is summarized in Table 2. The isolate was susceptible to ampicillin, chloramphenicol, erythromycin, tetracycline, and clindamycin, while exhibiting reduced susceptibility to vancomycin and streptomycin a profile consistent with intrinsic resistance patterns commonly reported for *L. plantarum*.^4,32^

**Table 2:** Antibiotic susceptibility of *L. plantarum* SG1 (Kirby Bauer disk diffusion)^a^

Antibiotic	Disc content (µg)	Inhibition zone (mm)	Interpretation
Ampicillin	10	28.4 ± 1.2	Susceptible
Chloramphenicol	30	26.1 ± 0.8	Susceptible
Erythromycin	15	22.7 ± 1.0	Susceptible
Tetracycline	30	25.3 ± 1.4	Susceptible
Clindamycin	2	19.6 ± 0.9	Susceptible
Vancomycin	30	18.2 ± 0.7	Reduced susceptibility (intrinsic)
Streptomycin	10	14.1 ± 0.9	Reduced susceptibility (intrinsic)

a Values represent the mean inhibition zone diameter ± SD.

### 3.12. Summary of one-way ANOVA results

A consolidated view of the F-statistics and corresponding probability values across all probiotic-functional parameters is provided in Table 3. In every quantitative parameter, the treatment factor (quercetin concentration) produced significant differences (*p* < 0.001), with F values ranging between 52.94 and 198.34 (auto-aggregation). Levene's test confirmed the homogeneity of variances (*p* > 0.05) for all variables, validating the use of one-way ANOVA without data transformation.

**Table 3:** Consolidated one-way ANOVA results for all studied parameters^a^

Parameter	*F* (df = 3, 8)	df_1_, df_2_	*p*
Acid tolerance	142.36	3, 8	<0.001
Bile salt tolerance	87.42	3, 8	<0.001
GIT survival	124.78	3, 8	<0.001
Hydrophobicity	156.89	3, 8	<0.001
Auto-aggregation	198.34	3, 8	<0.001
Co-aggregation	96.43	3, 8	<0.001
Co-aggregation	102.87	3, 8	<0.001
Co-aggregation	118.52	3, 8	<0.001
Co-aggregation	81.69	3, 8	<0.001
Inhibition zone	60.27	3, 8	<0.001
Inhibition zone	52.94	3, 8	<0.001
Inhibition zone	71.83	3, 8	<0.001
Inhibition zone	56.41	3, 8	<0.001
DPPH scavenging	134.27	3, 8	<0.001
ABTS scavenging	119.56	3, 8	<0.001

a All assumptions of one-way ANOVA (normality by Shapiro–Wilk test, homogeneity of variances by Levene's test) were verified and satisfied (*p* > 0.05). Where appropriate, Tukey's Honestly significant difference *post hoc* test was applied to identify pairwise differences.

## 4. Discussion

The present study provides an integrative evaluation of how quercetin, a representative dietary flavonoid, modulates the probiotic and functional properties of an indigenous *L. plantarum* SG1 isolate recovered from raw cow milk. The data demonstrate that sub-inhibitory quercetin concentrations, particularly 1/2 MIC, function as a physiological potentiator rather than as an antimicrobial stress agent: this concentration simultaneously improved gastrointestinal survival, surface adhesion traits, antimicrobial metabolite production, and radical scavenging capacity, without compromising the intrinsic safety profile of the strain.^3,5^

### 4.1. MIC determination and selection of sub-inhibitory doses

The MIC of quercetin against *Lactiplantibacillus plantarum* SG1 was 256 µg mL^−1^, and 1/2 MIC (128 µg mL^−1^) and 1/4 MIC (64 µg mL^−1^) were used as sub-inhibitory exposures. Across the measured phenotypes, 1/2 MIC produced the largest favorable changes, whereas responses declined at MIC. This biphasic pattern is compatible with a concentration-dependent stress response in which moderate polyphenol exposure may alter membrane organization, redox balance, or stress-response signaling without arresting growth, while a higher concentration imposes inhibitory stress. However, the present experiments did not directly measure membrane potential, permeability, ATP status, stress-response proteins, or gene expression. Accordingly, the 1/2 MIC response should be interpreted as an empirical phenotypic optimum under the tested conditions rather than as a molecularly established threshold.

### 4.2. Acid and bile salt tolerance

Survival under simulated gastric acidity (pH 2.5, 3 h) is considered a critical predictor of probiotic functionality.^24,33^ The 1.8-fold increase in survival observed in the 1/2 MIC group (58.4% *versus* 32.5% in the control) is mechanistically coherent: quercetin can act as a mild membrane-protective polyphenol, partially counteracting the dissipative effect of low pH on cytoplasmic homeostasis while avoiding complete inhibition of proton motive force.^17,34^ Importantly, these results exceed those of Huligere *et al.*, who^7^ reported acid tolerance values of 45–52% for *L. plantarum* isolates without polyphenol pretreatment, suggesting that the combination of an indigenous raw-milk isolate with sub-inhibitory quercetin produces a cumulative effect. The diminished viability at MIC (19.6%) parallels findings of dos Santos *et al.* and^16^ indicates that excessive polyphenol concentrations destabilize membrane lipids at very low pH.

Bile salt tolerance, another important component of probiotic screening, increased from 65.7% in the untreated group to 89.5% at 1/2 MIC quercetin and decreased to 52.1% at MIC. One possible explanation is that sub-inhibitory polyphenol exposure elicits adaptive changes in membrane homeostasis or general stress responses, whereas inhibitory exposure reduces the capacity to maintain these processes. Nevertheless, efflux systems, chaperones, bile-resistance genes, membrane composition, and intracellular ATP were not measured in this study; these mechanisms therefore remain hypotheses requiring direct validation.

### 4.3. Sequential gastrointestinal simulation

The 1/2 MIC group retained 48.9% viability after the sequential *in vitro* digestion procedure compared with 24.8% in the untreated control. This result supports improved *in vitro* stress tolerance under the tested conditions. It does not, however, establish efficacy in a food matrix or during refrigerated storage. No fermented-milk formulation, shelf-life study, sensory evaluation, or matrix-specific viability assessment was performed; consequently, claims regarding functional dairy product or synbiotic formulation development have been restricted to future application potential.

### 4.4. Cell surface properties and aggregation

The MATH assay evaluates bacterial hydrophobicity, a key determinant of adhesion to intestinal epithelial surfaces.^25,35^ The marked increase in hydrophobicity at 1/2 MIC (67.1% *versus* 42.3% in the control) supports the notion that quercetin, in sub-inhibitory concentrations, may interact with surface-exposed proteins or lipoteichoic acids and modulate surface charge without compromising cell integrity. Auto-aggregation, a predictor of colonization potential and barrier function, paralleled this trend.^26,27^ The decline observed at MIC reinforces the view that dose–response in polyphenol–bacteria interactions is non-linear: low doses can act as conditioning agents, whereas higher doses become inhibitory.^18,34^

### 4.5. Co-aggregation with pathogens

Co-aggregation is a mechanistic prerequisite for competitive exclusion of pathogens in the gut ecosystem. The 1/2 MIC group produced the strongest co-aggregation with all four indicator pathogens, with the highest value observed against *L. monocytogenes* (45.8%). This pattern not only mirrors the auto-aggregation data but also substantiates the hypothesis that quercetin-induced surface modifications promote pathogen recognition and binding.^17,34^ The relatively lower values observed against *Salmonella typhimurium* are consistent with the different surface chemistry of Gram-negative bacteria, in which lipopolysaccharide architecture limits hydrophobic interactions.

### 4.6. Antimicrobial activity of CFS

The widening of inhibition zones in the 1/2 MIC condition—for instance, against L. monocytogenes (22.4 mm *versus* 17.8 mm in the control)—is consistent with reports that sub-inhibitory polyphenol exposure can stimulate secondary metabolite production, including bacteriocins, organic acids, and low-molecular-weight antimicrobial peptides.^10,28,36^ The MIC-induced reduction (zones comparable to or smaller than those of the control) supports our interpretation that 1/2 MIC represents the optimal “sweet spot” for activating metabolite secretion without compromising biomass accumulation.^9,11^

### 4.7. Antioxidant activity

The DPPH and ABTS radical scavenging activities followed the same dose–response pattern as the other parameters. The 1.6- to 1.8-fold increase observed at 1/2 MIC is consistent with reports that *L. plantarum* can release low-molecular-weight peptides, exopolysaccharides, and short-chain fatty acids that act synergistically with polyphenols to neutralize radicals.^6,37^ The relatively higher values for ABTS compared to DPPH align with previous observations that the ABTS˙^+^ radical is more sensitive to hydrophilic scavenging due to its water-soluble nature.^29,30^ The decline observed at MIC reflects reduced metabolite secretion from metabolically stressed or dormant cells.

### 4.8. Safety profiling

The absence of hemolytic, DNase, and gelatinase activities is consistent with FAO/WHO and EFSA guidance on probiotic safety assessment. The antibiotic susceptibility pattern, with intrinsic reduced susceptibility to vancomycin and streptomycin, is the canonical phenotype of *L. plantarum* and has been documented in numerous previous studies.^8,14^ Quercetin supplementation did not alter any of these safety endpoints, suggesting that metabolic modulation at sub-inhibitory doses does not impose new toxicological liabilities.

### 4.9. Integration and implications

Taken together, the data show a reproducible concentration-dependent association between quercetin exposure and several measured probiotic-associated phenotypes of *Lactiplantibacillus plantarum* SG1. The novelty of the present work is therefore stated narrowly: it provides an integrated assessment of quercetin exposure in the specific indigenous SG1 strain isolated from raw cow milk collected in Erzurum Province, Türkiye, rather than claiming priority for polyphenol–lactic acid bacteria research in general. The observed peak at 1/2 MIC is a laboratory-defined phenotypic optimum and should not be interpreted as proof of a specific molecular regulatory mechanism. Translation to food applications requires validation in an actual dairy matrix and during storage.

### 4.10. Limitations

Several limitations should be considered. First, a matched DMSO-only vehicle control was not included, so solvent-related effects on bacterial surface properties, growth, or metabolite-associated readouts cannot be fully excluded. Second, although viable plate counts were used to calculate survival, replicate-level raw CFU mL^−1^ data are not reported and should be included prospectively alongside percentage survival. Third, no transcriptomic, metabolomic, proteomic, membrane-composition, or targeted biochemical analyses were performed; proposed mechanisms involving stress-response pathways, membrane protection, efflux, bacteriocin biosynthesis, or antioxidant metabolism therefore remain hypotheses. Fourth, no fermented-milk or other food-matrix validation, storage-stability assessment, or sensory analysis was conducted, so practical functional-dairy claims require further study. Finally, the *in vitro* gastrointestinal model does not reproduce the complete *in vivo* mucosal environment, immune interactions, or microbiota cross-talk.

## 5. Conclusions

The present study shows that quercetin exposure is associated with concentration-dependent changes in the probiotic-associated and functional characteristics of the indigenous *Lactiplantibacillus plantarum* SG1 strain isolated from raw cow milk. Under the tested *in vitro* conditions, 1/2 MIC produced the most favorable phenotypic profile, including higher acid and bile survival, gastrointestinal survival, hydrophobicity, aggregation, antimicrobial activity, and radical-scavenging activity, whereas MIC reduced most of these responses. These findings identify 1/2 MIC as an empirical optimum within the tested concentration range, but they do not establish the underlying molecular mechanism. Because no DMSO-only vehicle control, omics analysis, or food-matrix validation was included, solvent effects cannot be fully excluded and mechanistic and translational conclusions should remain cautious. The work therefore provides a basis for future studies using matched vehicle controls, transcriptomic/metabolomic or targeted biochemical analyses, absolute CFU reporting, and validation of SG1–quercetin combinations in fermented dairy matrices during storage.

## Author contributions

S. Akbulut: conceptualization, data curation, formal analysis, investigation, methodology, software, visualization, writing – original draft, writing – review and editing.

## Conflicts of interest

There are no conflicts to declare.

## Supplementary Material

RA-OLF-D6RA06744A-s001

## Data Availability

Summary data supporting the findings of this study are included in the article and its supplementary information. Replicate-level raw CFU mL^−1^ data are not available in the current dataset and have not been reconstructed. Supplementary information: supplementary numerical data for the probiotic and functional characterization of *Lactiplantibacillus plantarum* SG1, including acid tolerance, bile salt tolerance and simulated gastrointestinal survival (Table S1), co-aggregation with foodborne pathogens (Table S2), and antimicrobial activity/inhibition zone data (Table S3). See DOI: https://doi.org/10.1039/d6ra06744a.
