# Supplementary material for: Quercetin enhances the probiotic and functional characteristics of indigenous Lactiplantibacillus plantarum SG1 isolated from raw cow milk
Source: RSC Adv. 2026 Sep 8;16(43):52376–87. doi: 10.1039/d6ra06744a (PMC13551258; doi:10.1039/d6ra06744a)
Supplement: Supplementary file 1 [file RA-016-D6RA06744A-s001.pdf]

Supplementary Information (SI) for RSC Advances.
This journal is © The Royal Society of Chemistry 2026

Supporting Information

Quercetin Enhances the Probiotic and Functional Characteristics of Indigenous *Lactiplantibacillus plantarum* SG1 Isolated from Raw Cow Milk

Sumeyye Akbulut

This Supporting Information contains the numerical data corresponding to Figures 1–3 in the main manuscript, as requested by the reviewer.

Table S1. Effect of quercetin on acid tolerance, bile salt tolerance, and gastrointestinal survival of *L. plantarum* SG1.

**Parameter**	**Control**	**1/4 MIC (64 µg/mL)**	**1/2 MIC (128 µg/mL)**	**MIC (256 µg/mL)**	**F**	**p**
Acid tolerance (pH 2.5, 3 h)	32.5 ± 2.1^{a}	41.8 ± 2.6^{b}	58.4 ± 3.1^{c}	19.6 ± 1.8^{d}	142.36	<0.001
Bile salt tolerance (0.3%, 4 h)	65.7 ± 3.2^{a}	78.2 ± 3.0^{b}	89.5 ± 2.7^{c}	52.1 ± 4.1^{d}	87.42	<0.001
Overall GIT survival	24.8 ± 2.1^{a}	35.6 ± 2.5^{b}	48.9 ± 3.2^{c}	14.2 ± 1.9^{d}	124.78	<0.001

Note: Values are expressed as mean percentage survival ± SD (n = 3 biological replicates, each in triplicate). Different superscript letters (a–d) within the same row denote statistically significant differences according to one-way ANOVA followed by Tukey's HSD post-hoc test ($p < 0.05$).

Table S2. Effect of quercetin on co-aggregation of *L. plantarum* SG1 with foodborne pathogens.

Pathogen	Control	1/4 MIC	1/2 MIC	MIC	F	p
Escherichia coli O157:H7	22.4 ± 1.6^{a}	31.5 ± 2.0^{b}	39.6 ± 2.2^{c}	13.2 ± 1.4^{d}	96.43	<0.001
Staphylococcus aureus ATCC 25923	25.7 ± 1.8^{a}	33.8 ± 2.1^{b}	41.2 ± 2.3^{c}	14.8 ± 1.5^{d}	102.87	<0.001
Listeria monocytogenes ATCC 7644	28.9 ± 2.0^{a}	36.4 ± 2.2^{b}	45.8 ± 2.4^{c}	17.1 ± 1.7^{d}	118.52	<0.001
Salmonella typhimurium ATCC 14028	19.6 ± 1.4^{a}	27.1 ± 1.9^{b}	35.3 ± 2.0^{c}	12.4 ± 1.6^{d}	81.69	<0.001

Note: Values are expressed as mean percentage co-aggregation ± SD. Different superscripts indicate significant differences among groups (ANOVA + Tukey's HSD, $p < 0.05$).

Table S3. Antimicrobial activity (inhibition zone diameter, mm) of *L. plantarum* SG1 CFS against foodborne pathogens after exposure to quercetin.

Pathogen	Control CFS	1/4 MIC CFS	1/2 MIC CFS	MIC CFS	F	p
E. coli O157:H7	14.2 ± 0.9^{a}	17.6 ± 1.0^{b}	19.8 ± 1.1^{c}	13.5 ± 0.8^{a}	60.27	<0.001
S. aureus ATCC 25923	15.6 ± 1.0^{a}	18.4 ± 1.1^{b}	20.7 ± 1.2^{c}	14.9 ± 0.9^{a}	52.94	<0.001
L. monocytogenes ATCC 7644	17.8 ± 1.0^{a}	20.1 ± 1.0^{b}	22.4 ± 1.1^{c}	16.2 ± 0.9^{a}	71.83	<0.001
S. typhimurium ATCC 14028	13.4 ± 0.8^{a}	16.2 ± 0.9^{b}	18.6 ± 1.0^{c}	12.7 ± 0.8^{a}	56.41	<0.001

Note: Values are expressed as mean inhibition zone diameter (mm) ± SD, excluding the 6 mm well diameter. Different superscripts denote statistically significant differences ($p < 0.05$).
